# Supplementary material for: Targeting Pan-Cancer Stemness: Core Regulatory lncRNAs as Novel Therapeutic Vulnerabilities
Source: Int J Mol Sci. 2025 Dec 2;26(23):11684. doi: 10.3390/ijms262311684 (PMC12692698; doi:10.3390/ijms262311684)
Supplement: Supplementary file 1 [file ijms-26-11684-s001.zip › ijms-3978042-supplementary figures.pdf]

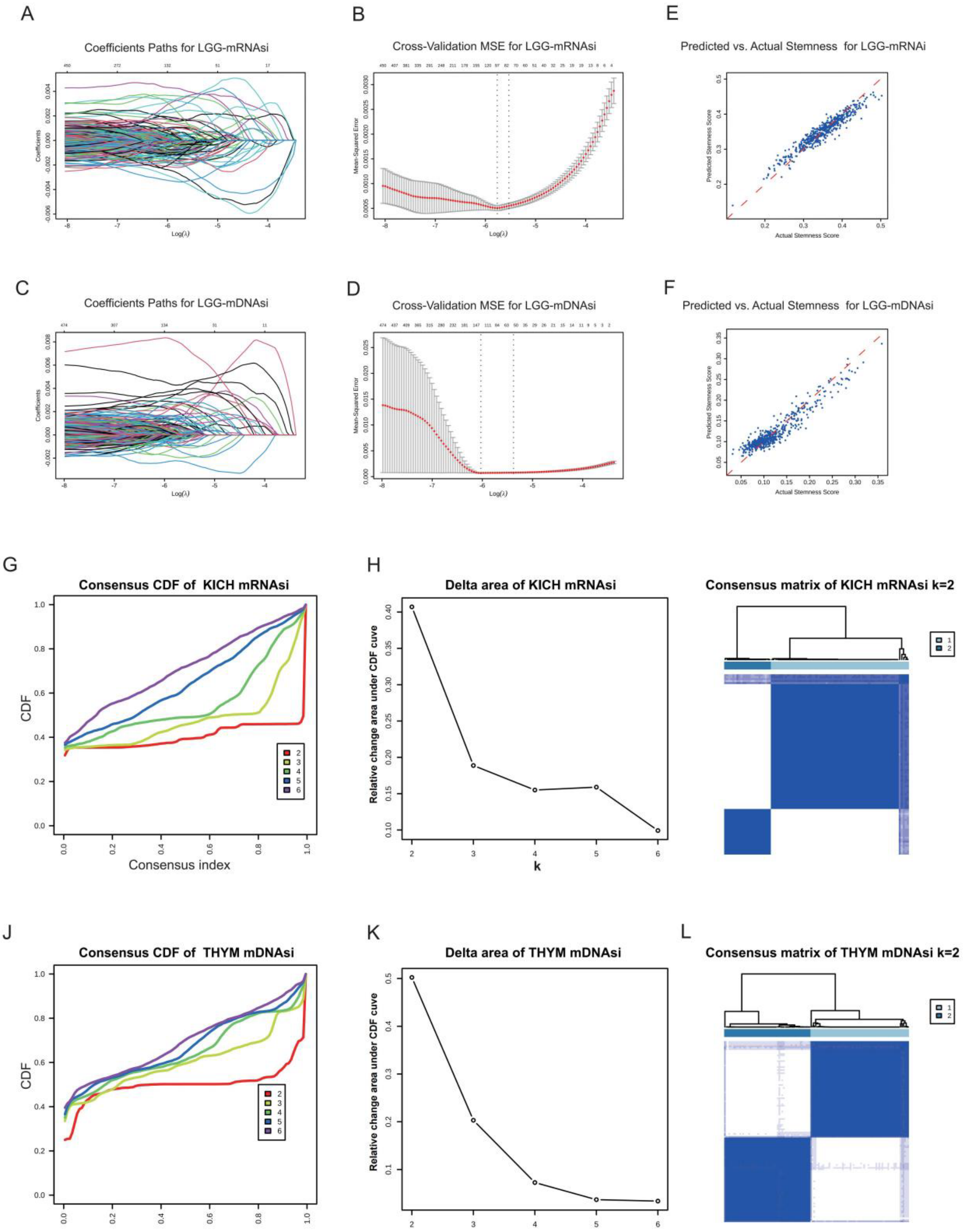


**Figure S1. Supplementary Figures for Methodology and Validation of lncRNA-based Stemness Analysis.**(A, C) LASSO coefficient paths for lncRNA selection, showing coefficient paths of lncRNAs in the LASSO regression model for the LGG-mRNAsi (A) and LGG-mDNAsi (C) datasets. (B, D) Optimal lambda selection by cross-validation, where Mean Squared Error (MSE) was plotted against the log(λ) parameter from a ten-fold cross-validation for the LASSO models in LGG-mRNAsi (B) and LGG-mDNAsi (D). The vertical dashed lines indicate the selected λmin and λ1se values. (E, F) Performance of the lncRNA-based stemness prediction models, illustrated by scatter plots showing the

correlation between the predicted stemness scores from the lncRNA-based LASSO model and the actual stemness scores for LGG-mRNAsi (E) and LGG-mDNAsi (F). The red diagonal line represents a perfect prediction (y=x). (H, K) Consensus clustering cumulative distribution function (CDF) curves for results with cluster numbers (k) ranging from 2 to 6. (I, L) Delta area plots for determining the optimal number of clusters, showing the relative change in the area under the CDF curve as k increases. The optimal cluster number K=2 was chosen for all cancer types.

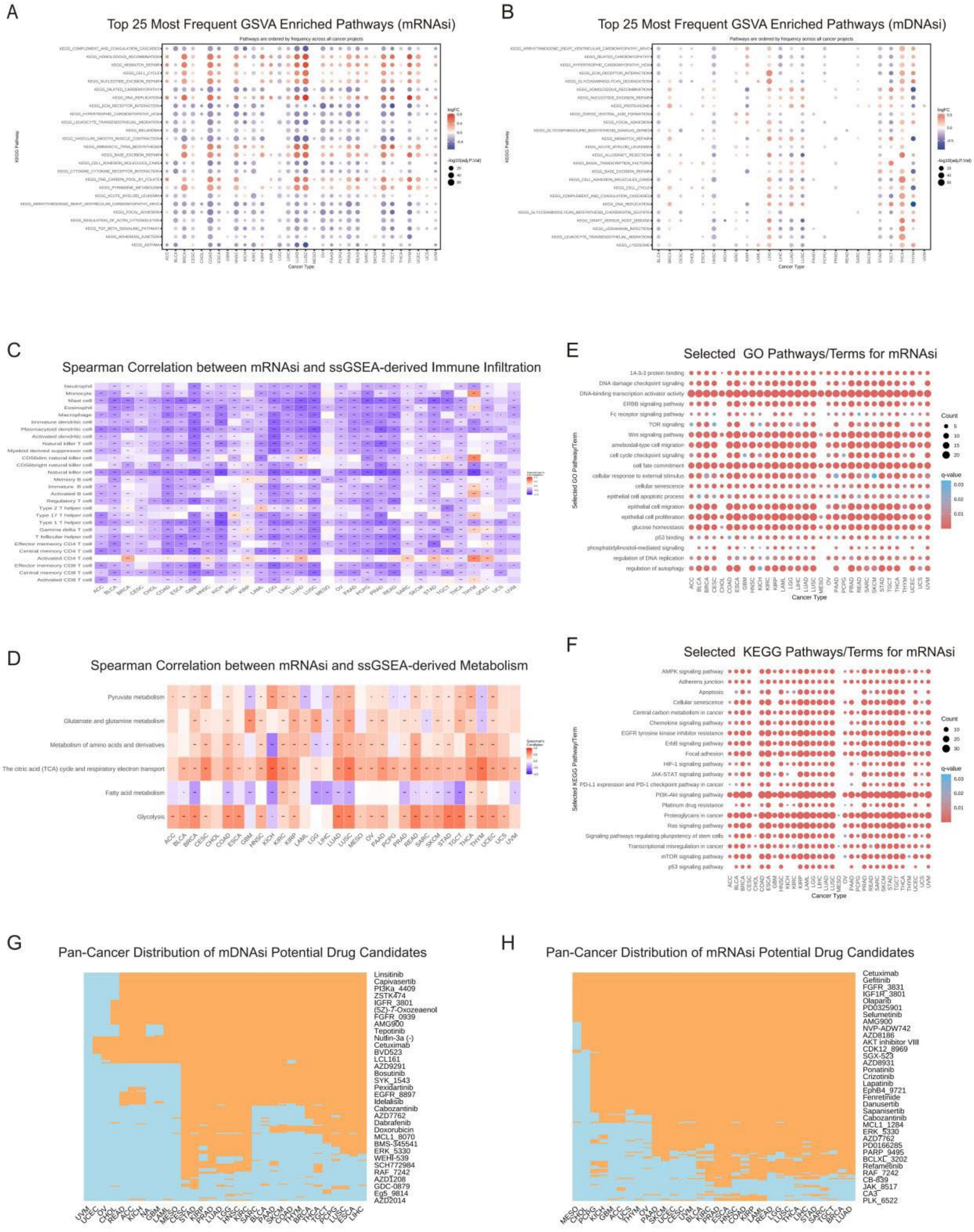


**Figure S2. Pan-cancer Multi-omics Characterization of Stemness Subtypes and lncRNA Networks.** (A-B)

Pan-cancer statistical analysis of GSVA enrichment scores. (A) Bar chart of the top 25 most frequent GSVA enriched pathways for mRNAsi , demonstrating consistent activation of pathways related to cell proliferation and genome maintenance in the High-stemness subtype. (B) Bar chart of the top 25 most frequent GSVA enriched pathways for mDNAsi. (C) Spearman correlation heatmap between the mRNAsi stemness index and ssGSEA-derived immune cell infiltration levels across pan-cancer types. The widespread negative correlations indicate that high-stemness tumors tend to exhibit "immune-cold" phenotypes. (D) Spearman correlation heatmap between the mRNAsi stemness index and ssGSEA-derived metabolic pathway activity. This analysis confirmed that the stemness index has significant positive correlations with pathways such as Glycolysis and Pyruvate metabolism. (E-F) Pan-cancer functional enrichment analysis of interacting proteins for stemness-related lncRNAs across multiple TCGA cancer types. (E) Gene Ontology (GO) enrichment analysis heatmap, showing that interacting proteins are significantly involved in processes like "DNA damage checkpoint signaling" and "cell cycle checkpoint signaling". (F) KEGG pathway enrichment analysis heatmap, confirming consistent activation of classical oncogenic pathways including "PI3K-Akt" and "mTOR" signaling pathways. (G-H) Heatmaps demonstrating the pan-cancer distribution of potential drugs associated with stemness-related lncRNA networks. (G) Distribution of potential drug candidates related to mDNAsi. (H) Distribution of potential drug candidates related to mRNAsi.

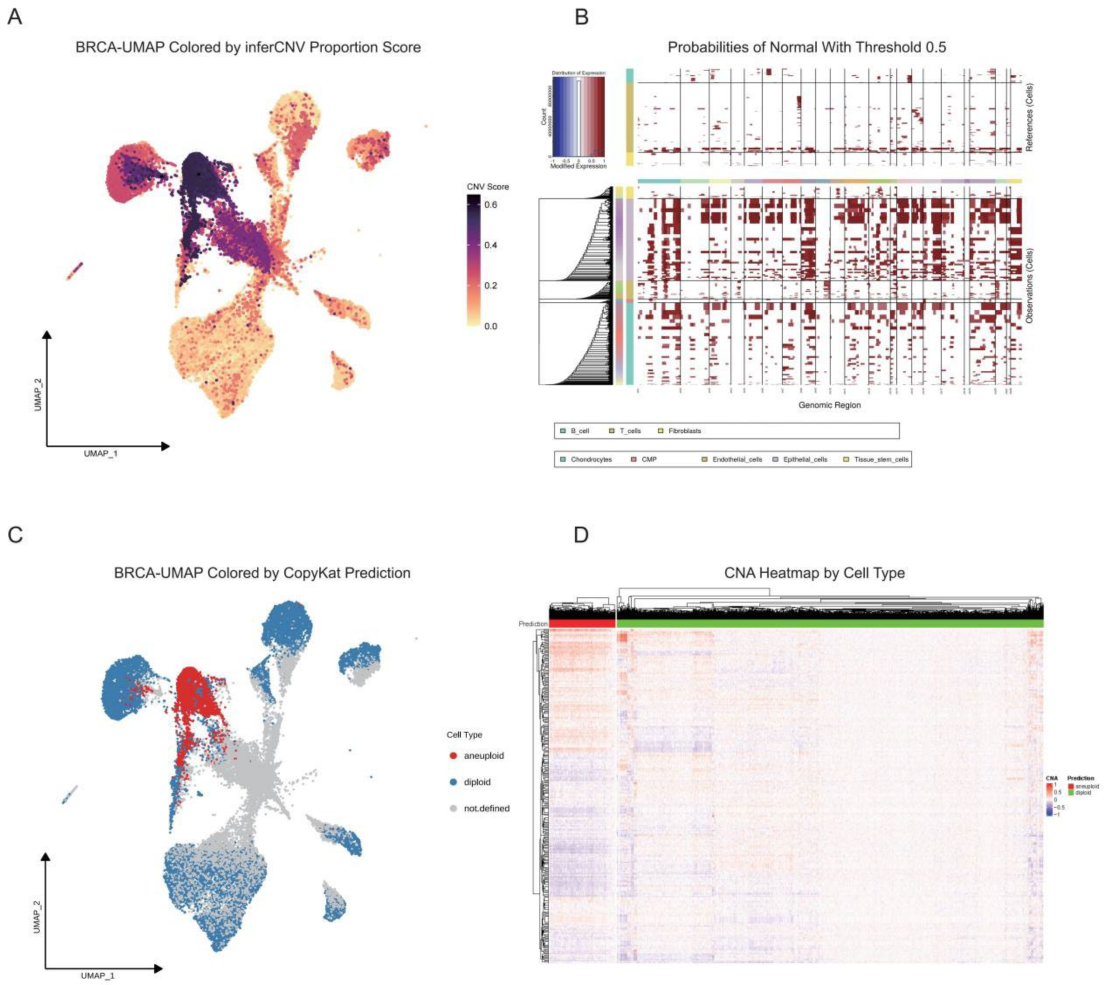


**Figure S3. Identification of Malignant Cells in BRCA Single-Cell Data using CNV Analysis.** (A) UMAP visualization of BRCA cells, colored by the inferCNV Proportion Score. Higher scores (darker colors) indicate a greater

degree of copy number variation. (B) Heatmap from the inferCNV analysis, illustrating inferred copy number alterations across genomic regions for reference cells (top panel) and observation cells (bottom panels). (C) UMAP visualization of BRCA cells, colored according to cell type predictions by the CopyKat algorithm. Cells are classified as aneuploid (red), diploid (blue), or not.defined (grey). (D) Heatmap showing Copy Number Alteration (CNA) profiles, with cells clustered by type. The top annotation bar corresponds to the CopyKat prediction (red: aneuploid, green: diploid), highlighting distinct CNA patterns between the predicted malignant and normal cells.

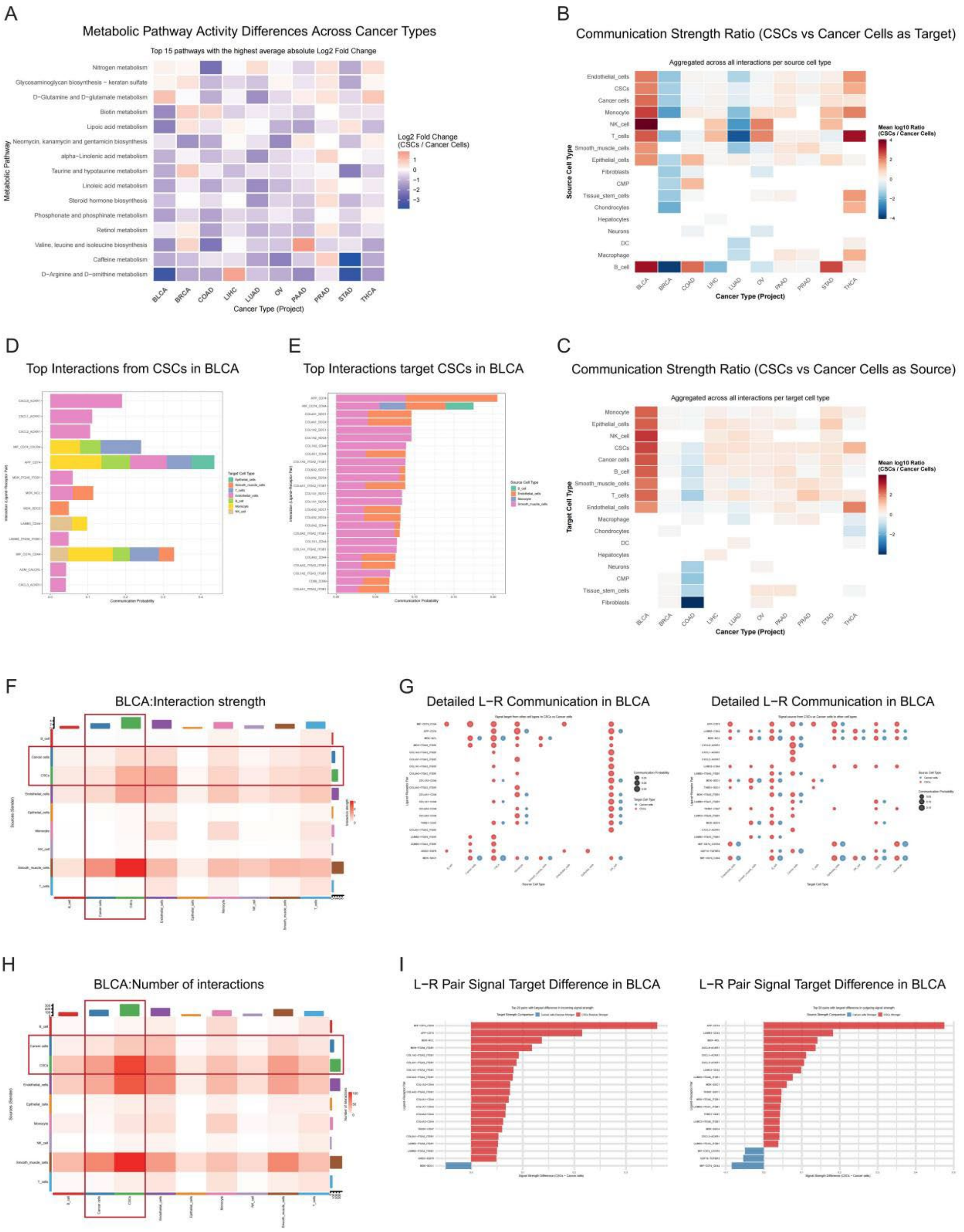

**Figure S4. Pan-cancer Functional and Communication Analysis of Cancer Stem Cells.** (A) Heatmap illustrating metabolic pathway activity differences across multiple cancer types. The map displays the top 15 pathways ranked by the highest average absolute Log2 Fold Change (CSCs vs. Cancer Cells). (B) Pan-cancer heatmap of the communication strength ratio (Mean Log2 Ratio, CSCs/Cancer Cells) with CSCs as the target, aggregated across all interactions per source cell type. (C) Pan-cancer heatmap of the communication strength ratio (Mean Log2 Ratio, CSCs/Cancer Cells) with CSCs as the source, aggregated across all interactions per target cell type. (D) Bar chart showing the top interactions originating from CSCs in BLCA. (E) Bar chart showing the top interactions that target CSCs in BLCA. (F) Heatmap visualizing the interaction strength between different cell types in BLCA. (G) Dot plots showing detailed ligand-receptor (L-R) communication in BLCA, including pathways like MIF-CD74_CD44. (H) Heatmap visualizing the number of interactions between different cell types in BLCA. (I) Bar plots showing the L-R pair signal target difference in BLCA, related to secreted signals such as CXC chemokines and MDK.

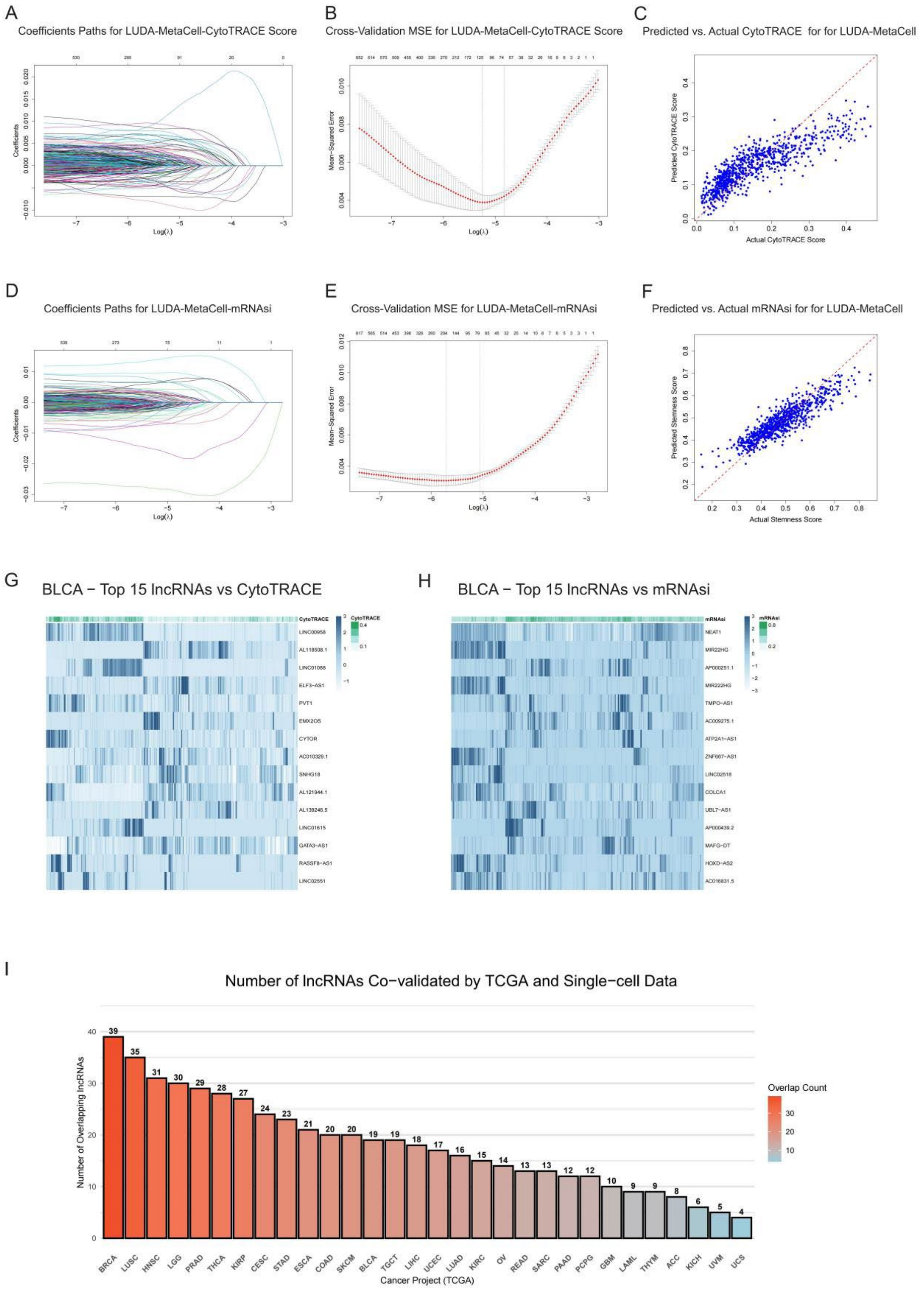


**Figure S5. Validation of lncRNA-based Stemness Models at the Single-Cell Level and Cross-Platform Comparison.** (A-C) Validation of the LASSO regression model for the LUDA-MetaCell-CytoTRACE Score. (A) Coefficient paths of lncRNAs in the LASSO model. (B) Ten-fold cross-validation Mean Squared Error (MSE) plot used for optimal lambda selection. (C) Scatter plot showing the correlation between predicted and actual CytoTRACE scores. (D-F) Validation of the LASSO regression model for the LUDA-MetaCell-mRNAsi. (D) Coefficient paths of lncRNAs. (E) Cross-validation MSE plot. (F) Scatter plot showing the correlation between predicted and actual

mRNAsi scores. (G-H) Heatmaps demonstrating the expression patterns of the top 15 lncRNAs most correlated with stemness scores in BLCA. (G) Top 15 lncRNAs correlated with the CytoTRACE score. (H) Top 15 lncRNAs correlated with the mRNAsi score. (I) Bar chart showing the number of overlapping stemness-related lncRNAs co-validated between TCGA bulk data and pan-cancer single-cell data. Bars are plotted for each TCGA cancer project, with color intensity corresponding to the overlap count.
